# Peripheral calcifying odontogenic cyst: a case report and comprehensive review of 60 years of literature

**DOI:** 10.3389/froh.2023.1223943

**Published:** 2023-08-04

**Authors:** Sally Sheng, Nicholas Tipton, Jennifer Chang, Hsiu-Wan Meng, Gena D. Tribble

**Affiliations:** School of Dentistry, University of Texas Health Science Center at Houston, Houston, TX, United States

**Keywords:** peripheral calcifying odontogenic cyst, oral pathology and oral medicine, case report, oral lesion, literature review

## Abstract

Peripheral Calcifying Odontogenic Cyst (PCOC) is the extraosseous form of calcifying odontogenic cyst that is limited to peripheral soft tissue without bony involvement. This case report presents a case of PCOC manifested as a progressive growth of gingival mass in a young male treated with excisional biopsy. Histological examination confirmed diagnosis of PCOC with presence of characteristic ghost cells and sporadic calcifications. No recurrence of the lesion and no complication were noted at three-year follow-up. Review of available literature on PCOC noted a predilection of occurrence in the mandible (61%) and in the anterior area of the jaws (58%). Mean age of patients was 41.7 ± SD25.43 (7–83) and 95% CI [33.6, 49.8] yrs. Mean size of the lesions was 1.38 ± SD1.1 (0.5–4.3) and 95% CI [0.93, 1.83] cm. Gender distribution was noted to be 51.3% male and 48.7% female.

## Introduction

Calcifying odontogenic cyst (COC) is a benign cystic neoplasm of odontogenic origin characterized by an ameloblastoma-like epithelium and ghost cells that have the potential to undergo calcification. COC was first described by Gorlin and colleagues in 1962 (1–3). The incidence of COC is estimated at 1%–3% of all odontogenic cysts and tumors (9, 37–41). Various classification systems and terminologies have been proposed in relation to COC due to ongoing debate as to whether COC is a neoplasm or a developmental cyst (4, 5). The World Health Organization (WHO) first described the lesion as non-neoplastic cystic lesion and used the term COC in 1971. In 1992, the WHO classified it as an odontogenic tumor but continued to use the term COC (5, 29). In 2005, WHO re-designated COC lesion as calcifying cystic odontogenic tumor (CCOT) (5, 29). In 2017, WHO changed the terminology back to COC and reclassified it as a benign odontogenic cyst (5, 29). COCs represent a heterogenous group of lesions that show a variety of clinicopathologic and behavioral features and may coexist with other odontogenic lesions (4, 5). Table 1 shows the various terminologies proposed for COC by different authors.

**Table 1:** Various terminologies of calcifying odontogenic cyst (4, 5).

Gorlin et al. (1962)	Calcifying odontogenic cyst (COC)
Gold et al. (1963)	Keratinizing Calcifying odontogenic cyst (KCOC)
Bhaskar (1965)	Keratinizing ameloblastoma (KA)
Fejerskov and Krogh (1972)	Calcifying ghost cell odontogenic tumor (CGOT)
Freedman et al. (1975)	Cystic calcifying odontogenic tumor (CCOT)
Praetorius et al. (1981)	Dentinogenic ghost cell tumor (DGCT)
Ellis and Shmooker (1986)	Epithelial odontogenic ghost cell tumor (EOGCT)
Toida (1998)	Calcifying ghost cell odontogenic cyst (CGCOC)
Colmenero et al. (1990)	Odontogenic ghost cell tumor (OGCT)
Buchner (1991)	Dentinogenic ghost cell tumor (DGCT) or EOGCT
WHO classification (1992)	Calcifying odontogenic cyst (COC)
Shear (1994)	Odontogenic ghost cell ameloblastoma (OGCA)
Wirshberg et al. (1994)	Odontocalcifying Odontogenic tumor (OOT)
WHO classification (2005)	Calcifying cystic odontogenic tumor (CCOT)
WHO classification (2017)	Calcifying odontogenic cyst (COC)

Since its introduction, terminology for COC continues to change throughout time due to a continued debate on whether there are two variants of the lesion - cystic and the neoplastic forms.

COC can present as intraosseous (Central Calcifying Odontogenic Cyst) or extraosseous (Peripheral Calcifying Odontogenic Cyst) lesion. The intraosseous central form has unicystic or multicystic radiolucent presentation, well defined borders, and is associated with bony destruction (1–3). The peripheral form (PCOC) represents less than 25% of reported COC cases (6). PCOC is less destructive and typically isolated peripherally in the adjacent soft tissues (6).

The main objective of this article is to discuss PCOC in a clinic case and review available literature to illustrate characteristics of PCOC, progression of changing terminologies, management of PCOC, and differential diagnosis of similar oral lesions.

## Case presentation

A 12-year-old Hispanic male patient presented with a chief complaint of a “progressive growth of gingiva” located palatal to the upper right lateral incisor. The patient reported that the lesion slowly increased in size over the span of six months and it was overall asymptomatic unless accidentally pressed then slight tenderness was reported. The patient denied any traumatic event relevant to the appearance of the lesion and denied any interference to eating, speech, or performance of oral hygiene. Due to the location of the lesion, the patient reported that he frequently pressed it with his tongue and would like to have it removed. Review of medical history was non-contributary. The patient was healthy without any medical conditions reported.

**Figure 1:**
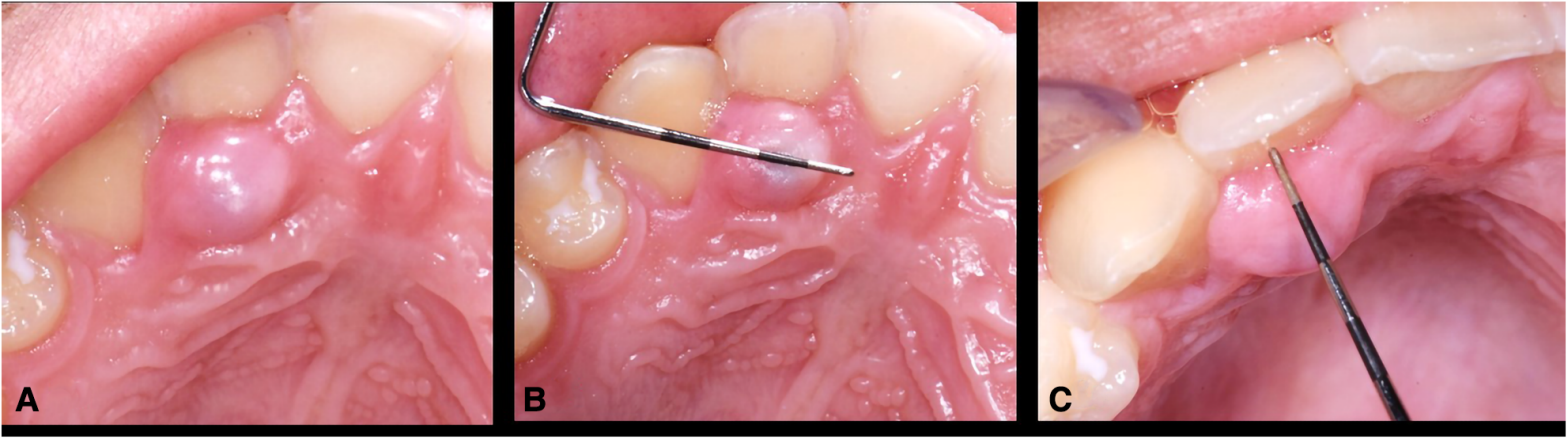
(**A–C**) Clinical presentation of the lesion. The 7 mm long × 9 mm wide × 5 mm high lesion over palatal gingiva of upper right lateral incisor had grown over the course of 6 months and became tender upon palpation. The lesion was generally firm but compressible pink to red, round, exophytic mass covered with normal keratinized tissue.

Clinical evaluation revealed a 7 mm × 9 mm × 5 mm convex, sessile, firm, depressible, round, pink, exophytic soft tissue lesion located along the palatal gingival margin of the upper right lateral incisor covered with normal keratinized tissue (Figures 1A–C). Periodontal probing noted 2–3 mm probing depth and vitality testing of surrounding teeth were normal. No displacement of teeth and no mobility of teeth were noted. Radiographic assessment noted for normal finding and ruled out endodontic lesions, bony involvement, or bony defects in the area.

**Figure 2:**
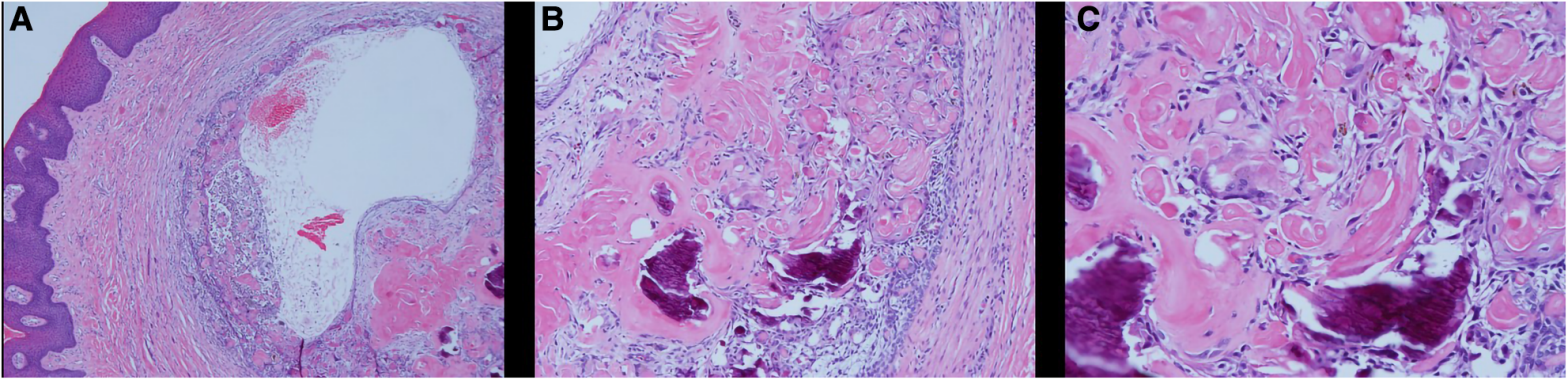
Histological examination of PCOC. Microscopic examination showing an oral soft tissue specimen with keratinized and non-keratinized epithelium with fibrous connective tissue. A cystic area in the fibrous connective tissue lined with well-defined layer of palisading ameloblastic-like basal cells, loosely arranged supra-basal epithelial cells resembling stellate reticulum, eosinophilic ghost cells devoid of nuclei, and basophilic calcification. Scattered chronic inflammatory cells were present. (**A**) HE × 4; (**B**) HE × 10; (**C**) HE × 20).

**Figure 3:**
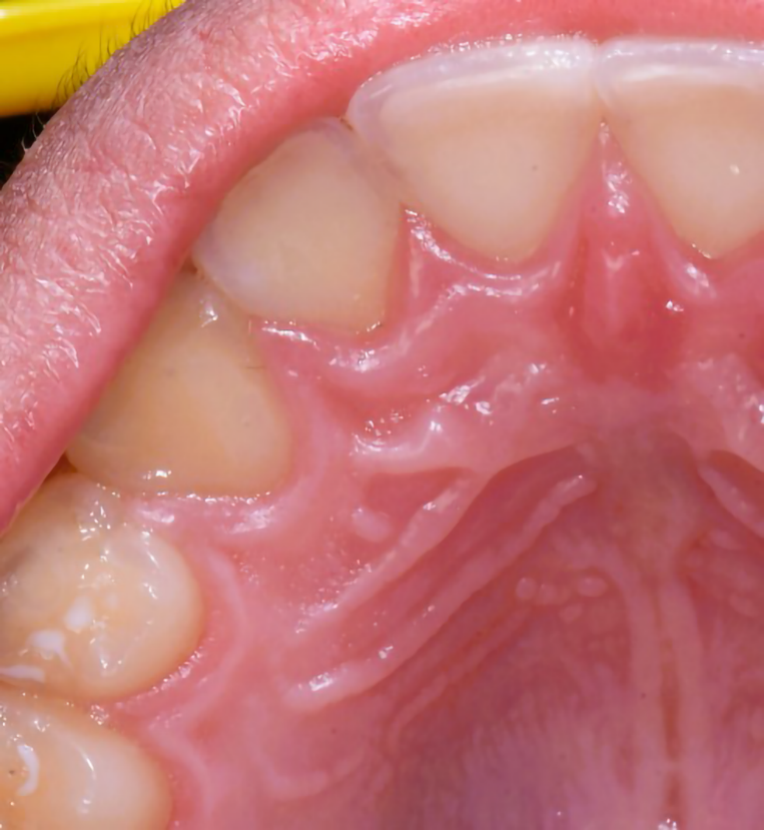
3-year follow-up of the area after excision of PCOC noted for normal healing and no recurrence of the lesion.

On the day of consultation, an excisional biopsy was performed. The lesion was excised in its entirety to bone and sent for histological examination. The patient was prescribed chlorhexidine oral rinse to be used twice a day for one week and over the counter ibuprofen was advised as needed for pain management. The microscopic examination revealed a well-defined cystic area lined with keratinized and non-keratinized epithelium with fibrous connective tissue. The cystic area in the fibrous connective tissue was lined with well-defined layer of palisading ameloblastic-like basal cells, loosely arranged supra-basal epithelial cells resembling stellate reticulum, eosinophilic ghost cells devoid of nuclei, and basophilic calcification. Scattered chronic inflammatory cells were present. A diagnosis of PCOC was confirmed microscopically and photomicrographs are presented in Figures 2A–C. Patient was seen two weeks later for post-operative check with normal healing noted. The patient reported uneventful healing without complications. The patient reported mild discomfort and minimal bleeding of the surgical site for 2–3 days after the procedure and no pain medication was needed post-operatively. At the 3-year follow-up, no recurrence of the lesion was noted and patient continued to report no concern or complication (Figure 3).

### Review of literature

A PUBMED search of case reports on PCOCs published between the years 1962 and 2023 was completed using the keyword: Peripheral Calcifying Odontogenic Cyst. A total of 52 publications were identified. All cases reviewed were published in English. Publications originally reported in a different language were excluded from our review. Review articles and meta-analysis were excluded due to the potential for subjects being accounted for multiple times and over-estimating the incidence of PCOC. Histological analysis and diagnosis of the lesions as PCOC using unique hallmarks of the lesion were required to be included in this study. 22 articles with total of 40 cases of PCOC were reviewed and the descriptive information from these cases and the current case are summarized in Table 2.

**Table 2:** Literature review of reported cases of peripheral calcifying odontogenic cysts.

Author	Case Number	Age/Gender	Race	Location	Size (cm)	Treatment	Duration/Recurrence (Y/N)/Follow-up m = month (s) year = year (s)
Swan (18)	1	9/M	Hispanic	Anterior maxilla	1	Excision	–
Claman (23)	2	7/M	White	Anterior maxilla	1	Excision	2 m/N/15 m
Hirshberg (24)	3	42/M	–	Anterior mandible	0.4–1	Excision	–/N/2year
Shamaskin (7)	4	63/M	Black	–	–	Excision	–
	5	52/F	Black	–	–	Excision	–
	6	15/M	White	–	–	Excision	–
	7	72/F	White	–	–	Excision	–
	8	67/F	White	–	–	Excision	–
Hong (25)	9	37/M	–	Anterior mandible	–	Excision	–
	10	61/M	–	Mandible	–	Excision	–
	11	74/F	–	Mandible	–	Excision	–
	12	76/M	–	Mandible	–	Excision	–
	13	49/F	–	Mandible	–	Excision	–
	14	50/F	–	Mandible	–	Excision	–
	15	79/M	–	Mandible	–	Excision	–
	16	69/F	–	Mandible	–	Excision	–
Castro (26)	17	83/F	Black	Anterior mandible	1–3	Excision	–/N/3year
Orsini (3)	18	39/M	–	Posterior mandible	–	Excision	12 m/N/–
Fregnani (15)	19	38/F	White	Anterior maxilla	1	–	–
	20	10/F	White	Anterior maxilla	1	–	–
Mesquita (17)	21	48/F	–	–	2	–	–
Manor (12)	22	72/-	–	Posterior maxilla	–	Excision	–
	23	72/-	–	Posterior mandible	–	Excision	–
Buchner (6)	24	12/F	White	Anterior maxilla	0.5–0.7	–	–
	25	76/M	White	Anterior mandible	0.5–0.7	–	–
	26	–/F	White	Anterior mandible	0.5–0.7	–	–
	27	–/F	Hispanic	Anterior mandible	0.5–0.7	–	–
	28	–/F	–	Posterior mandible	0.5–0.7	–	–
	29	–/M	–	–	0.5–0.7	–	–
Kumar (19)	30	40/M	–	–	0.5	Excision	–
Channappa (16)	31	30/M	–	Posterior maxilla	4.3 × 3.0	–	–
Reibel (21)	32	8/F	White	Anterior maxilla	1.0 × 1.5	Enucleation	3 m/N/–
Resende (2)	33	11/F	–	Posterior maxilla	–	Excision	5 yr/N/–
de lima (14)	34	39/M	–	Anterior maxilla	0.7	–	5 yr/N/–
Mittal (27)	35	17/M	–	Posterior mandible	1	Excision	4 m/N/1year
Sheikh (28)	36	65/F	–	Posterior mandible	2.5	Excision	3 m/N/1year
Costa (1)	37	9/M	–	Anteiror maxilla	1.0 × 0.5	Excision	36 m/N/–
Hsu (22)	38	48/F	–	Posterior mandible	–	Excision	60 m/N/–
Nahid (20)	39	24/M	–	Posterior maxilla	1.2 × 0.9	Excision	–
Oliveira (10)	40	10/M	–	Posterior mandible	<1.0	Enucelation	–
Current case	41	12/M	Hispanic	Anterior maxilla	0.7−0.9	Excision	6 m/N/3year
Total cases	41	Mean: 41.7 yrs SD 25.43 95% CI [33.6, 49.8] Female: 48.7% Male: 51.3%	White: 24.4% Hispanic: 7.3% Black:7.3% Unreported: 61%	Location not reported: 8/41 cases Maxilla:39% (13/33) Mandible:61% (20/4 = 33) Ant. Jaw: 58% Pos. Jaw: 42%	Mean: 1.38 cm SD 1.1 95% CI [0.93, 1.83]		No recurrence

Peripheral calcifying odontogenic cysts reported in the literature with their associated characteristics and information as reported in their original publications. 40 cases of PCOC were identified via PubMed search. Each row represents one subject, some had multiple subjects reported within a singular publication. Hashes (−) represent information about the subjects’ characteristics that were not reported. Age of patients and size of lesion are reported as mean ± standard deviation (range: min–max) and [95% confidence interval]. Mean age of patients was 41.7 ± SD25.43 (7–83) and 95% CI [33.6, 49.8] yrs. Mean size of the lesions was 1.38 ± SD1.1 (0.5–4.3) and 95% CI [0.93, 1.83] cm. Gender distribution was noted to be 51.3% male and 48.7% female. Predilection noted for occurrence in the mandible and anterior region of the jaws. No recurrence of the lesion after removal was reported.

## Discussion

Review of the 41 cases of PCOC reported noted a mean age of occurrence of 41.7 years old with standard deviation of 25.43 years and a range of 7 to 83 years. Gender distribution was noted to be 48.7% female and 51.3% male. 25 out of 41 cases did not report on the ethnicities of the patients. Of the cases that reported on ethnicities, 62.5% was White, 18.75% was Hispanic and 18.75% was Black. 8 out of 41 cases did not specify location of PCOC occurrence in their reports. Of the 33 cases that reported location of the lesion, a predilection was noted for mandible (61%). A predilection was noted for the anterior area (58%). The size of the PCOC reported had a mean of 1.38 cm (cm) with standard deviation of 1.1 cm and a range of 0.5 cm to 4.3 cm. The duration of the lesion before detection ranged from several months, to multiple years due to many being asymptomatic painless swelling. No recurrence was reported in any of the cases reviewed.

### Characteristics of PCOC

PCOC commonly presents as a uniform, painless, firm to soft gingival swelling without unique or distinguishing characteristics that would differentiate itself from other gingival pathologies commonly found in the oral environment. These lesions usually do not involve the underlying bone with very few rare cases reported with absorption of the adjacent alveolar bone, adjacent tooth root resorption, and potential for tooth displacement (1–3, 6, 7). PCOC lesions usually have no distinct radiographical characteristics, except in some case where sporadic calcifications in the lesions may appear on radiographs as radiopaque specks (6, 8). Histological analysis is often required for appropriate classification of these lesions. The most common histopathological findings of PCOC include a cystic lesion lined by epithelium with a well-defined ameloblastic-like basal layer of columnar cells in a palisaded fashion, an overlying layer with cells resembling stellate reticulum, and ghost cells that may be in the epithelial cyst lining or in the fibrous capsule (29). Presence of eosinophilic ghost cells is a characteristic microscopic feature of PCOC, however, may also be seen in other lesions, such as ghost cell odontogenic carcinoma, odontomas, and ameloblastic fibro-odontoma (29). Basophilic calcifications and calcifications of ghost cells can also be noted microscopically in PCOC lesions (29). The function of these ghost cells is currently debated but have been determined to not have a negative impact on the patient or with treatment outcomes (2).

Similar to previous study, our review found the most common site of occurrence for PCOC is the mandibular area, with a predilection for the anterior area of the jaw (2, 6). Previous studies found either similar distribution between genders or a higher percentage of occurrence in females while the present review noted a slightly higher percentage of occurrence in males (51.3%) (2, 6). The size of the PCOC lesions reviewed had a mean of 1.38 cm with standard deviation of 1.1 cm and a range of 0.5 cm to 4.3 cm.

### Management of PCOC

Due to their non-aggressive behavior, and low risk of recurrence, PCOC lesions are often treated with enucleation or excisional removal with favorable prognosis. Recurrence of PCOC after treatment has not yet been reported in literature. Excision can be done via conventional surgical blade, laser, or electrosurgery devices. PCOC is often limited in the peripheral oral soft tissue without bony involvement and has an average size of 1.38 cm, therefore complete removal of the lesion if often reported in literature. Alternative management methods of decompression, marsupialization may be considered in lesion of large size. The patient in the present case reported slow growth of the lesion over the span of six month. Due to the limited number of cases available in literature it cannot be concluded if left alone without treatment if PCOC would continue to slowly expand in size or self-arrest at some point. In addition to microscopic examination, it has been suggested that utilization of immunohistochemical investigations on cell proliferation activity may also aid in diagnosis of PCOC and may further contribute to further understanding on debate of its cystic or neoplastic nature (4).

### Progression of changing terminologies

In 1962, Gorlin first described the calcifying ghost cell odontogenic cyst (CGCOC) under the term calcifying odontogenic cyst (COC) (11). Since its introduction, its classification and terminology continue to change through time due to a continued debate on whether there are two variants of the lesion—cystic and the neoplastic forms (4, 5). The “monistic” concept believes that all COCs are neoplastic in nature. However, majority of COC are cystic in architecture and appear to be non-neoplastic. Hence, the “dualistic” concept believes that COCs exist as two entities: a cyst or a neoplasm (4, 5). A chorological timeline of the various classification and terminology systems are presented in Table 1 (4, 5).

### Differential diagnosis of oral peripheral soft tissue exophytic lesion

**Figure 4:**
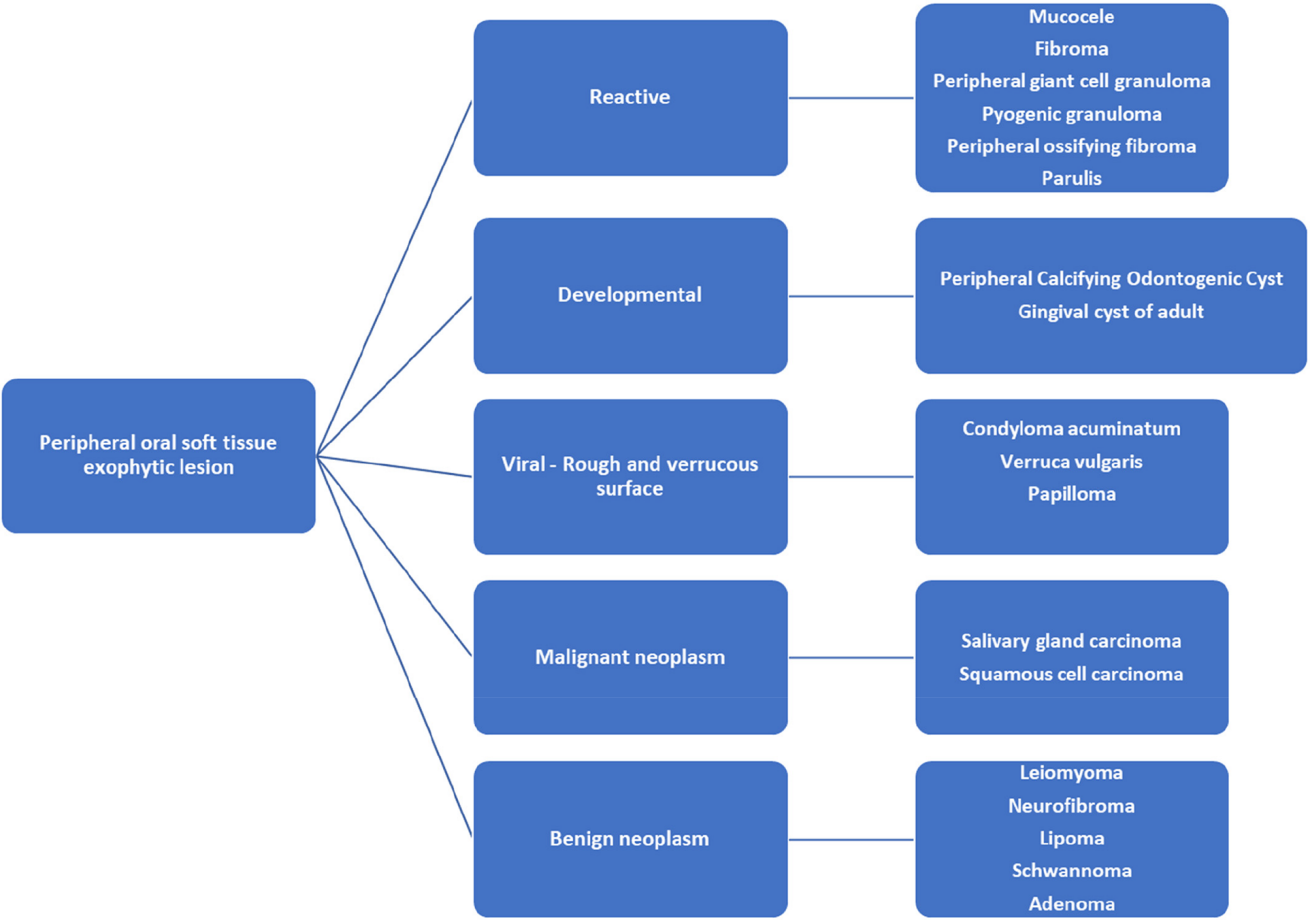
Differential diagnosis of oral lesion as soft tissue enlargement of the oral Mucosa (12, 13, 36).

Differential diagnosis of exophytic oral lesion with soft tissue enlargement can include various etiologies from reactive, viral, neoplastic, to developmental. Reactive lesions such as peripheral giant cell granuloma (PGCG) or pyogenic granuloma (PG) are inflammatory response to localized irritation or trauma with association to hormonal changes, or certain medications (30). PGCG often presents as a soft tissue purplish-red nodule consisting of multinucleated giant cells in a background of mononuclear stromal cells and extravasated red blood cells (30). PG often presents as smooth or lobulated exophytic lesion that is red-bluish in coloration and histologically noted for mass of angiomatous tissue with proliferation of capillary vessels and infiltrations of plasma cells, lymphocytes and neutrophils (31). Peripheral ossifying fibroma (POF) is a smooth surfaced reactive gingival enlargement to local irritant. POF often has normal squamous surface epithelium with proliferation of fibroblasts, endothelial proliferation, mineralized osteoids or calcifications, and inflammatory cells (34). Mucocele is a mucus retention lesion due to alteration to excretory duct of the salivary gland (35). It has smooth surface with normal coloration to bluish tint (35). Histologically, mucocele usually has hyperplastic parakeratinized stratified squamous epithelium and cystic space containing mucin and mucus-filled cells (35). Presence of salivary gland tissue and sialomucin is a diagnostic histological feature of mucocele (35). Traumatic fibroma usually presents as smooth-surfaced, normal-colored mucosa with sessile or pedunculated base as a reactive hyperplastic lesion to localized trauma (36). Histologically, irritation fibroma usually is noted as non-encapsulated nodular mass composed of fibrous connective tissue with collagen bundles interspersed with fibroblasts, blood vessels and scattered chronic inflammatory cells (36). The overlying squamous epithelium can be hyperkeratotic (36). Viral lesions such as condyloma acuminatum, verruca vulgaris, papilloma are associated with human papilloma virus (HPV) and have rough verrucous surface (32). Histologically theses lesions are noted for acanthosis with overlying hyperkeratosis and distinctive cells termed koilocytes (32). Koilocytes are large keratinocytes with abundant cytoplasm and small pyknotic nuclei often present in the upper layers of the epidermis (32). In addition to microscopic examination, in-situ hybridization, immunohistochemical studies for papillomavirus common antigen, or polymerase chain reaction may be used to confirm presence of HPV or further identify the HPV type (32). Developmental lesions may include PCOC as in this case or adult gingival cyst. The distinctive histological finding of PCOC is presence of ghost cells. Ghost cells are enlarged epithelial cells with eosinophilic cytoplasm and no nucleus which can be found in the lining epithelium and in the cyst lumen (1). Adult gingival cyst is a developmental odontogenic cyst that has mucosal surface lined by stratified squamous epithelium and a fibrous connective tissue capsule containing a cystic lumen lined by flat to cuboidal cells and mild chronic inflammatory cell infiltrates (33). Additional differential diagnosis of peripheral oral soft tissue enlargement may also include benign and malignant neoplasm. Differential diagnoses for oral soft tissue as exophytic lesion are listed in Figure 4 (12, 13, 36). Definitive diagnosis of peripheral oral exophytic lesions can be challenging and histological examination along with clinical correlation often is needed to aid in proper diagnosis.

### Limitations of present review

Literature on PCOC is limited to case reports and case series, which is considered lowest level of evidence in evidence-based research. Case reports and series can provide clinicians a useful narrative observational trend of the characteristic, incidence, location, and biological behavior of PCOC and gender, ethnicities, and age of patients. However, meaningful statistical analysis often is difficult with case reports/series due to limited number of case available in the literature and/or missing data. Data reported in case reports may not always be comprehensive and missing data can lead to missed observational trend. Ethnicity, gender, age of the patient, size, and location of the lesion were missing in some of the case reports reviewed. Incidence of PCOC may also be underreported due to the various terminologies and changing classification systems used throughout the years. This case report and review of literature presented a narrative description of the general characteristics on PCOC solely based on 41 case reports available in PubMed utilizing the key term PCOC. Systemic analysis of larger number of cases and inclusion of all available terminologies related to PCOC throughout the years in literature search key terms will provide a higher level of evidence of results.

## Data Availability

The original contributions presented in the study are included in the article/Supplementary Material, further inquiries can be directed to the corresponding author.
